# The sexual selection of creativity: A nomological approach

**DOI:** 10.3389/fpsyg.2022.874261

**Published:** 2023-01-09

**Authors:** Felipe Carvalho Novaes, Jean Carlos Natividade

**Affiliations:** Department of Psychology, Pontifical Catholic University of Rio de Janeiro, Rio de Janeiro, Brazil

**Keywords:** creativity, intelligence, sexual selection, proximate, ultimate, mating, ornament, WEIRD

## Abstract

Cultural innovations, such as tools and other technical articles useful for survival, imply that creativity is an outcome of evolution. However, the existence of purely ornamental items obfuscates the functional value of creativity. What is the functional or adaptive value of aesthetic and intellectual ornaments? Recent evidence shows a connection between ornamental creativity, an individual’s attractiveness, and their reproductive success. However, this association is not sufficient for establishing that creativity in humans evolved by sexual selection. In this critical review, we synthesize findings from many disciplines about the mechanisms, ontogeny, phylogeny, and the function of creativity in sexual selection. Existing research indicates that creativity has the characteristics expected of a trait evolved by sexual selection: genetic basis, sexual dimorphism, wider variety in males, influence of sex hormones, dysfunctional expressions, an advantage in mating in humans and other animals, and psychological modules adapted to mating contexts. Future studies should investigate mixed findings in the existing literature, such as creativity not being found particularly attractive in a non-WEIRD society. Moreover, we identified remaining knowledge gaps and recommend that further research should be undertaken in the following areas: sexual and reproductive correlates of creativity in non-WEIRD societies, relationship between androgens, development, and creative expression, as well as the impact of ornamental, technical and everyday creativity on attractiveness. Evolutionary research should analyze whether being an evolved signal of genetic quality is the only way in which creativity becomes sexually selected and therefore passed on from generation to generation. This review has gone a long way toward integrating and enhancing our understanding of ornamental creativity as a possible sexual selected psychological trait.

## Introduction

*“Sexual selection made our brains wasteful, if not wasted: it transformed a small, efficient ape-style brain into a huge, energy-hungry handicap spewing out luxury behaviors like conversation, music, and art.”* (Miller, 2000, p. 134).

Being creative secures undeniable practical benefits for survival. Crows and chimpanzees use twigs and create tools by modifying these twigs to better perform the desired aim (Reader et al., 2016). Chimpanzees, for instance, use such tools for termite fishing (Sanz et al., 2009). Humans have also created tools (e.g., handaxes) and various implements (e.g., clothes) that make it easier to get food and survive in diverse environments (Puccio, 2017). However, what would the evolutionary benefits be of body decoration, cave paintings, literary classics, philosophical treatises, or guitar solos?

**Figure 1:**
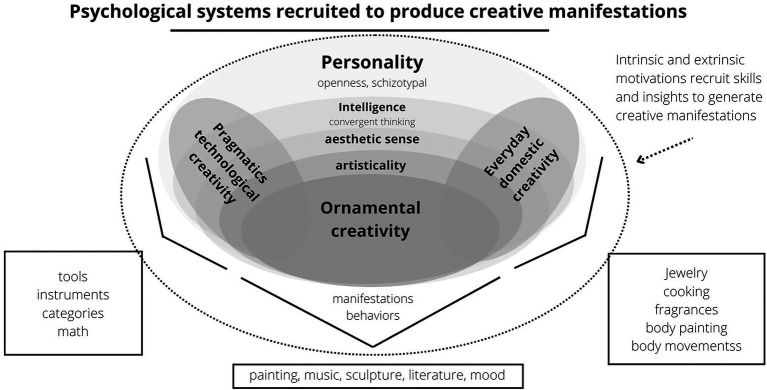
This scheme synthesizes the main variables associated with creativity and the possible relationships between them. A schematic illustration of a possible psychological structure of creativity. People who are more open to new experiences are more curious, flexible, and original, i.e., more creative and intelligent. Creativity involves divergent and convergent thinking, two characteristics associated with intelligence. Each type of creativity would operate according to its psychological mechanisms that are activated in the face of specific contexts and stimuli. These contexts and stimuli can have a reproductive nature, such as situations related to mate attraction or the visualization of a physically attractive partner. According to Feist (2001), ornamental creativity would have evolved by sexual selection because it was more conspicuous (e.g., making artistic pieces), just like the peacock’s plumage (Møller and Petrie, 2002, but also Askew, 2014 and Thavarajah et al., 2016), while technical creativity would have evolved by viability selection because it was more beneficial for survival (i.e., making tools). We also propose that reproductive motivations can mobilize everyday creativity; after all, people use their creativity and aesthetic sensitivity daily to beautify themselves (e.g., using makeup and clothes that enhance the most attractive features of the face and body, respectively; Stephen and Luoto, 2021; Varella et al., 2017; Valentova et al., 2022). Importantly, sexual selection would have shaped the psychological propensities to perform these manifestations and to enjoy them.

Darwin’s (1871) answer to that question was sexual selection. The ability to make tools must have evolved by viability selection, for it helped in survival in hostile environments, while the aesthetic skills required to create artistic performances and products would have evolved through sexual selection because they contributed to mate attraction. The extravagance of bird song and plumage, together with humans’ conspicuous drive to produce art and other forms of ornaments (e.g., self-grooming: Valentova et al., 2022; daily behaviors: Kapoor et al., 2021; and humor: Kaufman et al., 2007) would have a common evolutionary root (Darwin, 1871). The aesthetic sensibility, artistic capacities (including musicality), creative capacities, and creative motivation necessary to produce and appreciate (two different traits) these aesthetically conspicuous manifestations (e.g., art, music, paint, dance, humor, and metaphors) constitute a mental trait named *ornamental creativity* (Figure 1).

Recent studies seem to confirm that more creative people, particularly in the ornamental/aesthetic aspect, are considered more attractive in Western, educated, industrialized, rich, and democratic societies (see Karamihalev, 2013; Lebuda et al., 2021). Nevertheless, more than that is needed to show that creativity evolved by sexual selection.

A complete explanation must consider an ethological analysis, which holistically synthesizes evidence regarding mechanisms, ontogeny, phylogeny, and evolutionary function (Tinbergen, 1963), as well as a nomological network based on theoretical, cross-cultural, hunter-gatherer, phylogenetic, genetic, psychological, medical, and physiological evidence (Schmitt and Pilcher, 2004). The more evidence in that nomological network, the greater the chances that a mental trait is a psychological adaptation (Schmitt and Pilcher, 2004). Psychological adaptations are cognitive modules evolved to solve problems recurring in the environment of evolutionary adaptedness of a species (Schmitt and Pilcher, 2004). The consequences of these modules are flexible and adaptive behaviors, which allow them to be inherited by future generations as tendencies to develop the same modules ontogenetically (Schmitt and Pilcher, 2004). However, modules do not fossilize. How, then, to recognize them?

Human psychological adaptations can be recognized by criteria such as high efficiency, high complexity, high modularity, low phenotypic variance, low genotypic variance, low heritability, universality across cultures and individuals (Miller, 2000). However, the criteria used to identify adaptations evolved by viability selection differ from those used to identify adaptations evolved by sexual selection (Miller, 2000). Some of the ornamental adaptations evolved by sexual selection are fitness indicators. Effective fitness indicators are costly and wasteful, like a peacock’s plumage (Miller, 2000; Møller and Petrie, 2002; Askew, 2014; Thavarajah et al., 2016). Effective fitness indicators are also simple because they do not need to convey much information or to recruit many resources from the organism; they only need to “create a discriminable signal perceivable at a reasonable distance that reliably indicates a single quantity” (Miller, 2000). Fitness indicators must be sensitive to the covariation of different capacities to indicate their quality level, such as a peahen that notices a peacock’s exuberant tail and becomes inclined to mate with it (Miller, 2000). Furthermore, fitness indicators tend to vary more in the population than adaptations shaped by viability selection (Miller, 2000). That greater variability allows individuals to be sexually selected according to the display of the most costly indicator (Miller, 2000).

This review integrates existing evidence to explore whether creativity is or is not a psychological adaptation evolved through sexual selection. There are earlier attempts to review evidence about the evolution of mental abilities (Miller and Todd, 1998; Klasios, 2013), including creativity (e.g., Karamihalev, 2013), but these earlier attempts do not use the nomological approach presented here (Miller, 2000; Schmitt and Pilcher, 2004; Lewis et al., 2017).

Before proceeding, we must make some critical caveats. We do not suppose that creativity has evolved *only* by sexual selection. The idea is that the selection of creative partners has possibly overemphasized this ability, allowing it to be co-opted for ornamental purposes (Miller, 2000, 2001; Winegard et al., 2018). We are also not assuming that a trait evolved by sexual selection originated from sexual selection. Traits can initially evolve by viability selection and then be co-opted and exapted by sexual selection (e.g., foot fetish or bodily piercings; Luoto, 2019a). We are also not saying that sexual selection is only about sex differences (see Hooper and Miller, 2008; Janicke and Fromonteil, 2021), nor only male-biased sex differences (Miller, 2013; Varella et al., 2014, 2017; Rosenthal and Ryan, 2022). Further, sexual selection is not a proximate motivation (e.g., Bach could have been religiously motivated when composing music but still could have increased mating success because of his musical success; see Miller, 2000; Varella et al., 2013). Also, sexual selection is not only mate attraction but also relationship maintenance (long-term), intrasexual competition, intersexual conflict, and parenting (Kenrick et al., 2010; Petrie, 2021; Shuker and Kvarnemo, 2021). Furthermore, we are not claiming that ornamental creative ability is the only route to differential reproduction; other such domains include, but are not limited to, sports, physical enhancements, resource acquisition, parenting skills, and nepotism (Manning and Taylor, 2001; Stephen and Luoto, 2021; Walter et al., 2021; Varella et al., 2022).

## Definitions

Before approaching the multiple lines of evidence supporting creativity as a sexually selected trait, it is necessary to characterize creativity and its constituent variables (Figure 1).

### Creativity, capacity, and performance

Creativity is synonymous with originality and effectiveness (Runco and Jaeger, 2012). Creativity includes original aesthetic manifestations and precise imitations (non-original) of aesthetic manifestations. Creative people are often called innovative and inventive (Said-Metwaly et al., 2017). Research on creativity has focused on individual-level cognitive aspects as divergent thinking and intelligence (Runco and Jaeger, 2012; Karwowski et al., 2016b) and personality, mainly plasticity and openness (Karwowski et al., 2016b). From a process-focused perspective, creativity is a process of blind variation and selective retention of original ideas (Jung et al., 2013). Creative products result from these individual characteristics and processes and manifest in multiple domains, such as everyday, scholarly, performance, scientific, and artistic domains (Kapoor et al., 2021).

All of these creative domains are costly because successfully navigating them depends on healthy brains, and healthy brains are costly (i.e., energy-intensive and susceptible to instabilities throughout development; Miller, 2001). However, ornamental or aesthetic manifestations may be more expensive because they require a lot of energy expenditure without the practical benefits in return (from the point of view of survival; Feist, 2001). That would be the case for creativity employed in such domains as art and body beautification.

#### Divergent and convergent thinking

Creativity is often operationally defined as divergent thinking, although the connection between creative capacity, creative achievement, and divergent thinking is not always clear (Hornberg and Reiter-Palmon, 2017). Divergent thinking is the ability to come up with solutions, answers, or questions in response to an open problem of a visual or verbal nature (Hornberg and Reiter-Palmon, 2017). The level of divergent thinking depends on originality and fluency, where originality is the number of responses distinct from those of other individuals, and fluency is the overall number of responses (Hornberg and Reiter-Palmon, 2017). Divergent thinking alone does not guarantee creativity, and a dose of convergent thinking is also needed, which is defined as the ability to select the most helpful ideas from among those generated by the divergent associative process (Cropley, 2006).

### Personality

#### Openness, extroversion, and plasticity

Openness to experience and extroversion are the two personality components most consistently associated with creativity (Karwowski and Lebuda, 2016; Hornberg and Reiter-Palmon, 2017; Vartanian et al., 2018). Individuals higher in openness to experience are flexible, curious, less conventional, and tend to seek sensations and stimulation. The search for sensations and stimulation are occasionally pointed out as characteristics of extroversion as well (Karwowski and Lebuda, 2016). Openness and extroversion make up a second-order factor called *plasticity* in a model called Big Two, which positively predicts creativity and beliefs about creative capacity (Puryear et al., 2017; Feist, 2019). Openness to experiences is divided in sub-dimensions. The *intellect* sub-dimension, linked to intellectual curiosity, predicts success in science; the *openness* sub-dimension, linked to desire of adventure, predicts success in the arts (Feist, 1998).

#### Schizotypal and autistic traits

Schizotypy is a personality feature that seems to be associated with creativity. Schizotypal traits are characterized by positive symptoms, such as magical thinking, unusual perceptual experiences, impulsive nonconformity, and negative symptoms, such as introversion, emotional instability, and cognitive disarray (Holt, 2019). Schizotypy is positively related to originality and divergent thinking (e.g., Wang et al., 2018; Holt, 2019). Exaggerated schizotypy leads to exaggerated and unexpected associations, which leads to exaggerated creativity, observed mainly in artists (Acar and Sen, 2013; Carter et al., 2019; Aguilera and Rodríguez-Ferreiro, 2021). Artists often display “healthy schizotypy,” that is, higher creativity with no psychosis symptoms (Holt, 2019; Rantala et al., 2022). For example, poets tend to be more schizotypal, associating less obviously related ideas (Acar and Sen, 2013).

Autistic traits may also be associated with creativity (Baron-Cohen et al., 2001). Subjects with autism are inhibited in fluency and flexibility, but they have high levels of attention to details and originality; thus, subjects with autism are creative for different reasons, compared to the general population (for a meta-analysis, see Pennisi et al., 2020). Further, individuals with non-clinical autistic phenotype score lower on self-report creativity scales but exhibit greater creative performance in tasks involving drawing (Jankowska et al., 2019) and greater convergent thinking in anagram solution tasks (Abu-Akel et al., 2020).

### Intelligence

Intelligence is also involved with creativity. Intelligence (i.e., *g* factor or cognitive ability) is the capacity to think, plan, solve problems, and adapt to the environment (Cattell, 1963). More specifically, intelligence is a general factor that emerges from performance in specific and interrelated domains (e.g., verbal, spatial, mathematical). It comprises the ability to reason (fluid intelligence) and the ability to acquire knowledge (crystallized intelligence; Cattell, 1963; see also Kovacs and Conway, 2019).

Solving problems requires intelligence. However, it also requires originality and thinking beyond the obvious. Thus, it is difficult to distinguish between creativity and intelligence. Creative people also tend to be intelligent (Kim, 2008; Karwowski et al., 2021). For example, a meta-analysis that included 11,418 people showed that success in mathematics (a field with performance closely linked to IQ) is associated with creativity (Bicer et al., 2021). For instance, fluid intelligence and originality are positively correlated (Silvia, 2008). Higher fluid intelligence is associated with a higher use of metaphors (Silvia and Beaty, 2012). Individuals with better memory show more divergent thinking (Silvia and Nusbaum, 2013). Those with higher divergent thinking have higher verbal, figural (Cho et al., 2010), and visuospatial reasoning (Kell et al., 2013). In other words, intelligence is a necessary condition of creativity (Guilford, 1967).

These relationships can also manifest in specific fields. Scientists need to analyze problems systematically (which demands intelligence) to reach innovative outcomes (creativity; Karwowski et al., 2021). Elaborating disruptive theories requires “thinking outside the box” and pattern recognition (Feist, 1998). On the other hand, artists can work by associating ideas more freely (Boyd, 2010; Karwowski et al., 2021). Thus, intelligence and creativity are essential to the arts and sciences, but technical fields are more cognitively loaded than the arts (Feist, 1998; Park et al., 2007).

### Aesthetic sensibility

Aesthetic sensibility is the ability to assess the quality of sensory stimuli, such as abstract drawings and human faces. Eibl-Eibesfeldt (1989) describes three levels in human aesthetic psychology: (1) the basic level, which we have shared with most vertebrates and includes regularities, symmetry, harmony, and superstimuli; (2) the human-specific level, which is universal and relates to a human “sense of order” that underlies more specific attributes such as balance, rhythm, rhyme, and harmony; (3) the local culture level which is related to traditions and tastes shared within each human population.

The capacity to perform aesthetic appraisals (visual) is associated with intelligence, divergent thinking (figural but non-verbal), and personality (aesthetic openness; Myszkowski et al., 2014). Creative ornamental products are judged more on their aesthetic rather than technical merit, thus, they are expected to rely more on aesthetic sensibility (Feist, 2001). Personality also seems to be associated with aesthetic sensibility. People with greater openness to experience and schizotypy tend to appreciate artistic activities more (Feist and Brady, 2004) and have higher aesthetic motivation (Furnham, 2021).

It is necessary to emphasize that aesthetic and artistic sensibility are not synonymous. Esthetic sensitivity is a more general capability than artistic; the two are independent adaptations of each other (Varella et al., 2011). For example, many animals distinguish different types of human art; however, few animals find it as reinforcing as humans do (e.g., Varella, 2021), which points to the specificity of human artistic appreciation (Watanabe, 2013).

## Levels of analysis and nomological network of evidence

Why do human beings employ their creativity in making original works of art? In line with previous proposals, we believe that part of the answer concerns sexual selection (Darwin, 1871; Low, 1979; Eibl-Eibesfeldt, 1989; Zahavi and Zahavi, 1997; Miller and Todd, 1998; Miller, 2000; Miller, 2001; De Block and Dewitte, 2007; Baer and Kaufman, 2008; Dutton, 2009; Varella et al., 2011, 2017, 2022; Verpooten and Nelissen, 2012; De Ridder and Vanneste, 2013; Karamihalev, 2013; Westphal-Fitch and Fitch, 2018; Luoto, 2019a). However, to test this hypothesis, the evidence must be collected from diverse levels of analysis (mechanisms, development, stimuli, phylogeny, and function; Tinbergen, 1963; Varella et al., 2012; Fitch, 2015). More recently, this approach has been expanded by investigating cultural, social, biological, and environmental inputs that activate psychological modules (Lewis et al., 2017; Luoto, 2019a). To answer whether creativity evolved by sexual selection, one needs to establish whether different sources of evidence converge toward indicating that ornamental creativity serves reproductive ends (Table 1).

**Table 1:** Predictions based on sexual selection and supporting evidence.

Question	Yes	No
Is there genetic influences in creativity?	Reuter et al. (2006) – polymorphisms of the dopamine D2 receptor gene DRD2; mathematical talent and convergent thinking; Runco et al. (2011) – genes DRD2, DAT, COMT, DRD4, TPH1 for verbal and figural fluency; Zabelina et al. (2016) – COMT and DAT for good cognitive flexibility and medium top-down control; Mayseless et al. (2013) – 7R polymorphism in the dopamine receptor D4 gene DRD4 in participants with higher divergent thinking scores and particularly flexibility scores; Mosing et al. (2014) – associations between music practice and music ability were predominantly genetic; Tan et al. (2014) – gene AVPR1A and SLC6A4; Zwir et al. (2022) – modern humans have genetic basis for creativity that chimpanzee and Neanderthal do not	
Is individual differences in creativity partially heritable?	Coon and Carey (1989) – Yes, but the effects of common environment were almost always larger; Grigorenko et al. (1992) – 0.44 in creative thinking in adolescents; Velázquez et al. (2015) – creative personality 50%–54%; Velázquez et al. (2015) – 38%–47% in creative drawing; Vinkhuyzen et al. (2009) – 60% in arts; Vinkhuyzen et al. (2009) – 83% in creative writing; Piffer and Hur (2014) – 61% in creative achievement; Kandler et al. (2016) – 62% in perceived and 26% figural creativity; Piffer and Hur (2014) – 43%–67% in creative achievement; Roeling et al. (2017) – 67% in the choice of artistic professions and of 43% in scientific professions; Mosing et al. (2015) – 51% among women regarding musical aptitude; 57% for men, and 9% for women in musical achievement; Mosing et al. (2014) – music practice was substantially heritable, 40%–70%	
Are androgens (T) associated with creativity?	Fukui (2001) – music listening decreased T in men and increased in women; Hassler (1992) – the better the musical performance, the wider the T range; Crocchiola (2014) – both male and female artists had significantly lower 2D:4D ratios, i.e., more T; Sluming and Manning (2000) – musical ability within the orchestra were associated with lower male 2D:4D, i.e., more T; van der Linden et al. (2020) – positive association between T indicators, intelligence and scientific achievement; Iijima et al. (2001) – girls with congenital adrenal hyperplasia make drawings typical of normal boys; Kanazawa (2000) – The productivity of single scientists takes longer to drop compared to married ones, and being married is associated with a drop in T	
Is there sexual dimorphism in creativity?	Baer and Kaufman (2008) – sex differences in specific domains of creativity; Beaussart et al. (2012) – men have a higher drive for creative display; Cheung and Lau (2010) – girls in the junior high grades excelled boys in verbal flexibility, figural fluency, figural flexibility, figural uniqueness, and figural unusualness; Ellis et al. (2008) – meta-analysis showing more precursors of appreciation art related traits in women/girls and precursors of production art-related traits in men/boys; Greengross et al. (2020) – meta-analysis showing that men have higher humor production ability and women have higher humor appreciation; He (2018) – female superiority in creative thinking-drawing during childhood and early adolescence; He and Wong (2011) – girls outperformed boys in thoroughness of thinking, boys outperformed girls in boundary-breaking thinking; Hemdan and Kazem (2019) – females’ creative performance was significantly better than males’ in the Creativity Index score; Hora et al. (2021) – meta-analysis showing male advantage in creative performance; Lange (2011) – men are more prone to verbal display production than women; Lange and Euler (2014) – most literature is produced by men of reproductive age, in a sample with 18th-, 19th-, and 20th-century writers; Low (1979) – ornamentation occur in both sexes, according with social status, wealth and power; Nakano et al. (2021) – Most studies reported gender differences, with 45.20% in favor of women, 23.28% in favor of men, and 31.50%, oscillating according to the content evaluated; Savage et al. (2015) – predominance of male music performances; Varella et al. (2017) – review showing higher women’s inclination toward artistic domains; Varella et al. (2022) – women showed higher score in Visual arts and Musical arts, while men scored higher in Literary arts, Sport, and Circus arts; Varella et al. (2010) – women like to sing and men like to play musical instruments	Baer and Kaufman (2008) – review showing no sex differences in creative ability or creative achievement in general; Varella et al. (2010) – no sex difference in the amount to music listened per day
Is there only greater male variability in creativity?	He (2018) – greater male variability in China; He et al. (2013) – greater male variability in adolescents in China; He and Wong (2011) – greater male variability in China	Karwowski et al. (2016b) – greater male variability in originality and unconventionality and greater female variability in adaptiveness; Karwowski et al. (2016a) – higher variability of creative ability between males and females in Meru, Kenya; He et al. (2015) – greater female variability in young children and greater male variability in young adults; Ju et al. (2015) – The greater male variability hypothesis in creativity is generally supported., but is inconsistent across samples; Lau and Cheung (2015) – both male variability and female variability increased with time, according to the responses to both verbal and figural stimuli; Taylor and Barbot (2021) – no differences in drawing, writing and divergent thinking in american men and women adults and adolescents
Is creativity attractive in other cultures?	Buss and Barnes (1986) – WEIRD and non-WEIRD cultures; Kamble et al. (2014) – India; Li et al. (2011) – United States and Singapore; Souza et al. (2016) – Brazil; Chang et al. (2011) – China; Varella et al. (2022) – Brazil and Czech	Lebuda et al. (2021) – Meru
Does creativity increase attractiveness?	Madison et al. (2018) – mate value ratings were generally increased by MPQ for raters of both sexes; May and Hamilton (1980) – men were found to be more attractive when paired with specific music styles; Marin and Rathgeber (2022) – male faces paired with music were considered more attractive; Marin et al. (2017) – women, but not men, gave significantly higher ratings of facial attractiveness and dating desirability after having listened to music than in the silent control condition; Watkins (2017) – male creativity impacted attractiveness more than facial beauty; Gao et al. (2017) – Male faces paired with novel metaphorical compliments were rated as more attractive by women than those paired with literal ones; Greengross et al. (2020) – women prefer men high in production ability and men prefer women high in appreciation ability; Lange et al. (2014) – the main effect of verbal proficiency on attractiveness was supported	Bongard et al. (2019) – results show that musicians’ profiles were not generally rated as more attractive than non-musicians; Lebuda et al. (2021) – creative potential negatively predicted the number of offspring
Is there an association between short-term sexual strategy (e.g., number of sex partners) and creativity?	Beaussart et al. (2012) – the link between creative activity and number of sexual partners was only significant for males; Clegg et al. (2011) – more successful male artists had more sexual partners than less successful artists but this did not hold for female artists; Nettle and Clegg (2006) – unusual experiences and impulsive nonconformity positively predicted the number of partners, when mediated by creative activity; Clegg et al. (2011) – more successful male artists had more sexual partners; Harrison and Hughes (2017) – greater musical ability had higher sex/drugs milieu scores, especially in women; Lange and Euler (2014) – literature production was correlated with number of mates; Mosing et al. (2015) – men with higher scores on the music achievement scale had more children; Varella et al. (2022) – positive association between literary arts, short-term mate value and sociosexual desire; women showed positive association between musical arts, short-term mate value; in men, circus arts were positively predicted by short-term mate value; White et al. (2018) – pursuing a short-term mating strategy was associated with selecting more atypical flirting behaviors	Harrison and Hughes (2017) – musicians and non-musicians do not differ in the number of sex partners; Varella et al. (2022) – in women, literary arts were not predicted by sociosexual behavior and not by the number of short-term relationships; in women, visual arts were not predicted by sociosexual behavior, attitude or desire and not by the number of short-term relationships; in women, musical arts were not predicted by sociosexual behavior and desire and not by the number of short-term relationships; in women, circus arts were not predicted by sociosexual behavior and desire and not by the number of short-term relationships; in men, literary arts, musical arts, circus arts and visual arts were not predicted by sociossexuality and also not by the number of short-term relationships
Is there an association between long-term sexual strategy and creativity?	Gao et al. (2017) – compliments on appearance using novel metaphors were preferred by women in a long-term relationship during the fertile phase; Kennair et al. (2022) – humor is more effective as flirtation when used by men in long-term context; Madison et al. (2018) – women’s preference for long-term relationships increased in the face of exposure to better musical performance quality; Mosing et al. (2015) – males and females who scored higher on the musical aptitude or music achievement measures scored lower on sociosexuality; Prokosch et al. (2009) – men perceived as more intelligent are more desirable for long-term relationships; Varella et al. (2022) – in women, engagement in literary art was negatively correlated with SOI-Attitudes; in women, engagement in visual arts was predicted positively by parenting effort; women showed that musical activities were predicted negatively by SOI-Attitudes; in men, esthetically enhance bodily movements are positively related to the number of long-term partners; in men, literary arts were predicted positively by long-term mate value	Varella et al. (2022) – in women, literary arts were not predicted by sociosexual behavior and not by the number of long-term relationships; in women, visual arts were not predicted by sociosexual behavior, attitude or desire and by the number of long-term relationships; in women, musical arts were not predicted by sociosexual behavior and desire and not by the number of long-term relationships; in women, circus arts were not predicted by sociosexual behavior, desire but were by the number of long-term relationships; in men, literary arts, musical arts, circus arts and visual arts were not predicted by sociossexuality and also not by the number of long-term relationships
Is there an association between ovulatory phase, production and appreciation of creative manifestations?	Charlton (2014) – woman only preferred composers of more complex music as short-term sexual partners when conception risk was highest; Haselton and Miller (2006) – fertile women prefer creative over wealthy men for short-term relationship; Galasinska and Szymkow (2022) – women ideas were the most original during the phase of ovulation; Galasinska and Szymkow (2021) – positive correlation between the probability of conception and both creative originality and flexibility; Gao et al. (2017) – compliments on appearance using novel metaphors were preferred by women in a relationship during the fertile phase; Miller et al. (2007) – female dancers earned more tips in the ovulatory period, which suggests an increase in aesthetic sense and/or creativity to perform more seductive movements	
Does mating motives enhance creativity?	Griskevicius et al. (2006) – short-term or a long-term mating goal increased creative displays in men, but in women, only long-term mating goal increase creative displays	
Is creative tendencies spontaneous, precocious and intrinsically motivated?	Amabile and Gitomer (1984) – extrinsic motivation decreases children’s creative performance; Bispham (2009) – intrinsic motivation makes people engage in music very early; Frois and Eysenck (1995) –creative and aesthetic capacities does not need artistic training; Morris (1962) – anedotic evidence that extrinsic motivation decreases creative performance of a chimpanzee; Trevarthen (1999) – early motivated to draw and dance; Varella (2021) – greater intrinsic motivation in students of artistic areas	
Does creativity and other g-loaded traits indicate genetic quality?	Arden et al. (2009a) – intelligence was a significant positive predictor of six of the eight abnormality counts, controlling for life style; Arden et al. (2015) – genetic relationship between intelligence and lifespan; Gajos and Beaver (2017) – Paternal age at birth appears to have a marginally significant nonlinear relationship with male children’s verbal IQ scores; Banks et al. (2010) – meta-analysis showing that smarter people are a bit more symmetrical; Spencer et al. (2005) – adult male canaries, Serinus canaria, infected with malaria, Plasmodium relictum, as juveniles, develop simpler songs as adults compared to uninfected individuals, and exhibit reduced development of the high vocal center, HVC, song nucleus in the brain; Mosing et al. (2015) – in women, there was a positive correlation between musical aptitude and music achievement with genetic quality measures; in male, only between musical aptitude and general intelligence	Arslan et al. (2014) –higher paternal age at offspring conception did not predict offspring intelligence; DeLecce et al. (2020) – no evidence for a relationship between intelligence and ejaculate quality; Garamszegi et al. (2018) – the study did not find statistical evidence for MHC allelic diversity being related to either the estimates of song output and complexity or syllable composition
Does other species manifest creative behaviors and dispositions?	Bandini and Harrison (2020) – review of innovation in chimpanzee; Catchpole (1987) – review showing song birds as a trait evolved by sexual selection; Endler (2012) – according to some definitions of art, Great Bowerbirds are artists, judge art, and therefore have an aesthetic sense; Garamszegi et al. (2018) – males performing song bird in sexual selection; Kawase et al. (2013) – male puffer fishes construct large geometric circular structures on the seabed that played an important role in female mate choice; Lefebvre (2013) – review of innovation and intelligence in birds and primates; Lefebvre et al. (2004) – review of innovation and intelligence in birds and primates; Macdougall-Shackleton (1997) – review showing song birds as a trait evolved by sexual selection; Taylor (2014) – review showing that corvids have complex cognition, use tools and think in complex ways; Reader and Laland (2001) – evidence that individual variation in the propensity to innovate in terms of sex, age, and social rank in primates; van Schaik et al. (2016) – orangutans seem innovative only or mostly in captivity	
Does creativity (and other g-loaded traits) generate reproductive success in other species?	Coleman et al. (2007) – mimetic vocalizations accuracy predicted male mating success; Boogert et al. (2011) – review indicating sexual selection of cognitive traits in non-human vertebrates; Catchpole (1987) – males with more elaborate songs attract females before males with lesser elaborate songs; Chen et al. (2019) – female budgerigars shifted their preference to previously non-preferred males after these males demonstrated the ability to solve a problem that stumped the originally preferred males; Keagy et al. (2009) – problem-solving ability predicts mating success; Macdougall-Shackleton (1997) – review showing that song bird contributes to sexual selection as well peacock’s tail; Minter et al. (2017) – females preferred mating with males who had better initial inhibitory control, a proxy for intelligence; Östlund-Nilsson and Holmlund (2003) – females were more attracted to males with nests containing sticks and spangles than to males with undecorated nests; Shaw et al. (2019) – superior male memory performance was associated with efficient offspring provisioning; Spritzer et al. (2005) – males with better spatial ability had larger home ranges and made more visits to different nestboxes than did males with poorer spatial ability	
Do human ancestors show sexual dimorphism in relation to creative manifestations?	Snow (2013) – persons who made hand stencils in the caves were predominantly females	Mackie (2015) – hand sprays were created by children and adults of both sexes suggesting non-exclusivity in activities associated with rock art creation; Rabazo-Rodríguez et al. (2017) – 11 hands belong to women and 10 to men

## Mechanisms

### Genetic

There are polymorphisms in alleles of genes associated with dopaminergic systems, such as genes DRD2, DAT, COMT, DRD4, and TPH1, which are also associated with creative abilities and achievements (e.g., Reuter et al., 2006; Runco et al., 2011; Mayseless et al., 2013; Zabelina et al., 2016; Luoto, 2019b). Such genes also appear to play a role in sexual selection. For example, D4 dopamine receptor gene variation is linked to infidelity and sexual promiscuity (Garcia et al., 2010; Acevedo et al., 2020). Furthermore, genes associated with creativity and preference for creative partners are correlated, which shows that creativity has been subject to sexual selection at some level, perhaps by assortative mating (Verweij et al., 2014).

The selection of these genes linked to creativity has an ancient history. Modern humans have over 200 unique non-protein-encoding genes that regulate the co-expression of many other protein-encoding genes in coordinated networks underlying modern capabilities such as creativity, which are not found in chimpanzees or Neanderthals (Zwir et al., 2022).

These genes provide a part of the biological substrate necessary for creativity; however, they do not necessarily imply the development of creative capacities. Inheriting the propensity to develop the ability differs from having the ability, which depends on adequate stimulation throughout ontogenetic development as well as a host of other neurodevelopmental and biopsychosocial factors.

### Neurotransmission and endocrine mechanisms

Dopamine has a role in creativity and sexuality, which suggests a link with sexual selection (Garcia et al., 2010; Acevedo et al., 2020). Dopaminergic activity is also related to psychomotor agitation behaviors such as eye blinking, that is a known clinical marker of accelerated dopaminergic activity observed in schizophrenic patients and non-clinical creative individuals (Akbari Chermahini and Hommel, 2009).

Testosterone is also related to creativity. For instance, men and women usually present a peak of musical talent from puberty (Hassler, 1992). Testosterone seems to enhance musical performance up to a certain level, but performance drops above this level (Hassler, 1992). Hassler (1992) conjectures that the positive effect of the hormone on musical creativity is mediated by its influence on spatial reasoning. In fact, children trained in a musical instrument have better indicators of intelligence and creativity (Benz et al., 2016; but see Mosing et al., 2014 for a discussion of genetic and practice effects). Artists of both sexes have a lower 2D:4D ratio, which suggests these individuals sustained more influence of testosterone during intrauterine development (Sluming and Manning, 2000; Crocchiola, 2014). Furthermore, there is a positive correlation between lower 2D:4D, amount of Nobel prizes, and scientific publications (van der Linden et al., 2020). On the other hand, concentrations of salivary testosterone and preference for sophistication levels of music are negatively correlated (Doi et al., 2018).

Hormonal influence in creative expressions can be detected in the patterns of drawings made by boys and girls. For instance, between 5 and 6 years old, boys draw more objects, use fewer colors and prefer cool colors, while girls draw more people and flowers, using more colors (Abraham, 2016). Girls with congenital adrenal hyperplasia (who consequently produce more androgens than the female average) present drawing patterns more akin to those made by boys (Iijima et al., 2001).

Though women with high testosterone display intense creative activity, the pattern seems more consistent in men. The masculine peak of artistic output happens in married men from 30 to 40 years old, extending beyond 40 for singles (Kanazawa, 2000). Men write 10 times more books and other literary outputs, accounting for more entries in the *Guinness World Records* (Lange, 2011; Lange and Euler, 2014).

So what, after all, is the role of testosterone in creativity? Androgen levels may be positively linked to increased performance and personality. For example, increasing performance in skills such as spatial reasoning and increasing willingness to take risks, compete, and seek novelty (Feist, 2019; Luoto and Varella, 2021). That would partially explain the male prominence in scientific and artistic fields, even without differences in creativity averages between the sexes (see Goldin and Rouse, 2000; Mosing et al., 2015).

### Neurobiological

Could brain architecture influence creativity and thus affect mate selection? More creative people exhibit brain laterality leaning to the right; however, the findings are mixed. For example, more schizotypal people use the right hand less (Somers et al., 2009) and show greater left asymmetry in the use of the senses (Lindell, 2014), which suggests greater right lateralization of the brain hemispheres. Whereas the left hemisphere is associated with access to more specific semantic networks, the right hemisphere is connected to more diffuse networks, connecting more general ideas in the semantic network, which may explain the activation of this hemisphere during divergent thinking (Lindell, 2014).

Lateralization of the brain hemispheres appears to be associated with testosterone. For example, boys with higher testosterone levels at puberty show greater right brain lateralization (Beking et al., 2018). Boys with higher levels of intrauterine testosterone showed the opposite lateral (i.e., left) activation at puberty (Beking et al., 2018), which seems more associated with an autistic profile (Castelli et al., 2002).

### Social mechanisms

#### Universal preference for creative partners

If creativity evolved through sexual selection, it would logically follow that creative partners will be in higher demand. Studies have shown that creativity is a trait desired in romantic partners by Americans (Buss, 1989; Buss et al., 1990; Li et al., 2011), Brazilians (Souza et al., 2016; Novaes, 2022), Chinese (Chang et al., 2011), Singaporeans (Li et al., 2011), Indians (Kamble et al., 2014), and others (Buss et al., 1990; Watkins, 2017; but see Lebuda et al., 2021). The importance given to creativity varies. Creativity, especially of the ornamental/aesthetic type (Kaufman et al., 2016; see Figure 1), starts to matter in mating as soon as primary preferences (such as physical beauty) are satisfied (Li et al., 2002). Creativity is so essential in attractiveness that it can make someone more attractive than social status (Buss and Barnes, 1986; Novaes, 2022), physical appearance (Watkins, 2017), or intelligence (Prokosch et al., 2009).

#### Creativity keeps partners together

The power of creativity can explain the universal preference for creative partners in partner retention. Couples that engage in novel and stimulating activities become closer (Aron et al., 2005). Thinking about romantic relationships stimulates creativity more than thinking about casual sex (Campbell and Fletcher, 2015). However, the consequences change depending on the type of creativity considered. For example, while everyday creativity increases romantic love, artistic creativity decreases it – but they did not elaborate explanations for these results in evolutionary terms (Campbell and Kaufman, 2017).

### Psychopathological mechanisms

**Figure 2:**
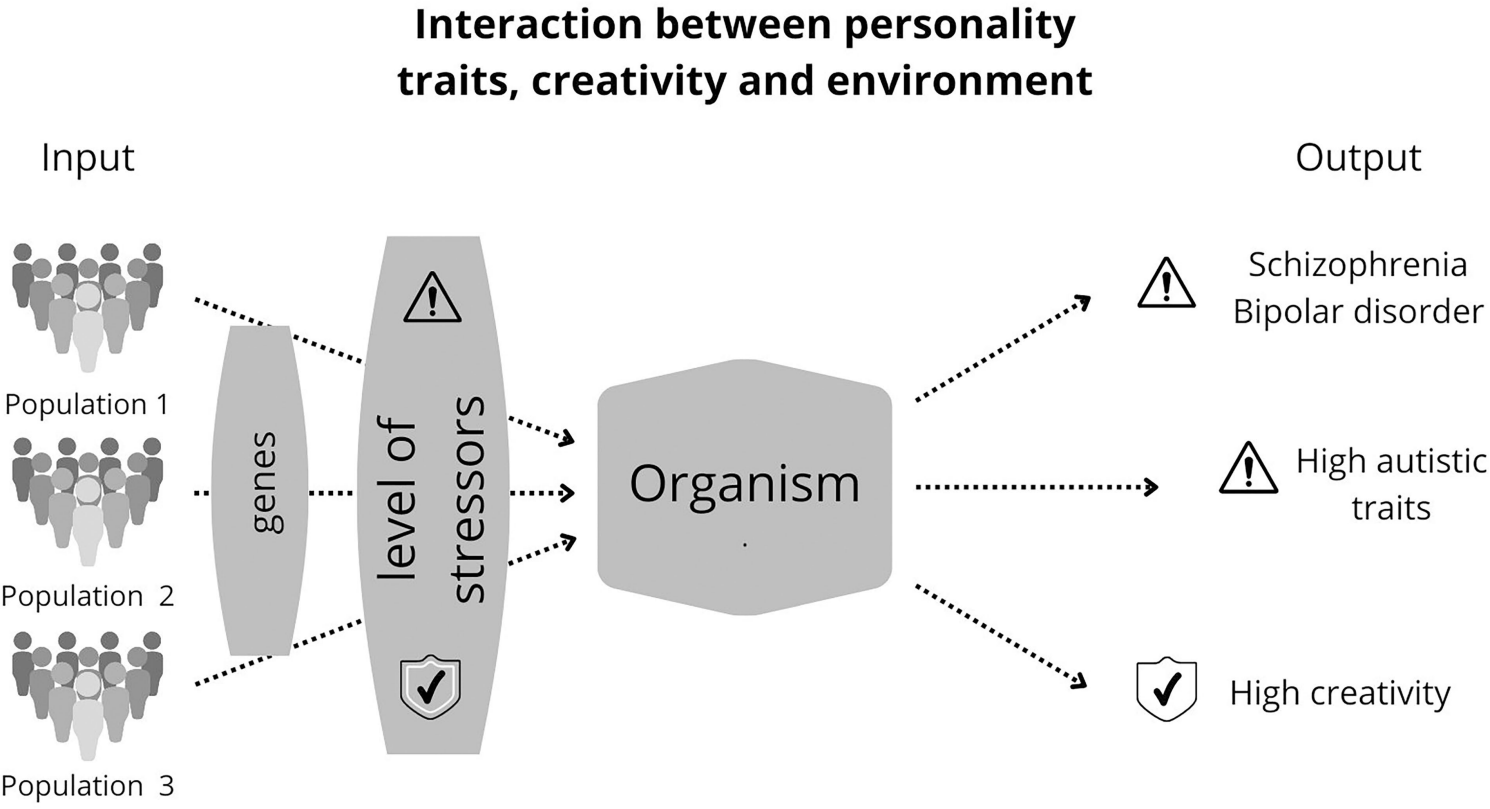
Consequences of stress on people with schizotypal and autistic traits. Creative people are moderately schizotypal. In other words, schizotypal people are often imaginative, associating ideas in unusual ways. People with schizophrenia and bipolar disorder also have these characteristics, sometimes manifesting them in a “dysfunctional way,” e.g., in a paranoid way, but not in an artistic way. That “dysfunctional manifestation” of creativity can occur because of stressors that disrupt normal nervous system development. Thus, individuals with the same genetic propensities for high creativity may manifest it in the form of a disorder or the neurotypical form, depending on how much the stressors have affected ontogeny. This figure shows three examples of populations, each subjected to different levels of environmental stress. The symbol with “!” means high levels of stressors, while the symbol “ok” means tolerable levels of stressors, that is, those that do not significantly impair neural development.

Fitness indicators are subject to instability in their development, affecting their carriers’ reproductive success (Klasios, 2013). For example, peacocks may have trouble developing their extravagant plumage because of deleterious mutations, environmental stress, or parasites (Møller and Petrie, 2002; Askew, 2014; Thavarajah et al., 2016). Likewise, developmental problems can impair brain development and the display of mental and, by extension, creative ornaments (Miller and Todd, 1998; Shaner et al., 2004; Del Giudice et al., 2010). Some psychological disorders may result from ontogenetically or evolutionarily disturbed mental adaptations (Figure 2; Rantala et al., 2019, 2021, 2022).

Schizotypal traits are linked to mental ornaments that involve creativity and can be displayed in various ways, such as verbally and visually (Shaner et al., 2004). Dysfunctional levels of schizotypy characterize schizophrenic people, and they suffer from verbal difficulties, such as a disorganized expression of thoughts through language (Shaner et al., 2004). Despite high levels of schizotypy, schizophrenic and bipolar people have difficulty expressing creativity (Acar and Runco, 2012; Acar et al., 2018). The association between schizotypy and creativity follows an “inverted U” shape, growing to a point beyond which one decreases while the other increases (Abraham, 2014). That explains the ambiguous findings on disorders and creativity (Acar and Runco, 2012; Acar and Sen, 2013; Acar et al., 2018).

Mood disorders are also associated with creativity only to a certain extent. A meta-analysis showed that (verbal) creativity and bipolarity are positively associated (Taylor, 2017). Epidemiological analyses reveal a higher prevalence of bipolar disorder among academics and artists (Kyaga et al., 2011). Likewise, people with a high expression of autistic traits are more prevalent in fields such as engineering and mathematics (Morsanyi et al., 2012). However, the association between creativity and success declines as the severity of psychopathological symptoms increases (Pennisi et al., 2020) – see Figure 2.

The shared genetic basis between mental disorders, personality, and creativity can explain these associations. In other words, the genetic risk of developing schizophrenia and bipolar disorder predicts artistic achievements positively (Power et al., 2015). More precisely, higher polygenic risk for bipolar disorder is associated with a higher tendency for divergent thinking (Takeuchi et al., 2021). Also, the polygenic risk of developing schizophrenia is positively associated with risk behavior (Li et al., 2020), which is linked to creativity (Feist, 2019). The same genetic component involved in verbal ability is involved in schizophrenia, which may explain verbal proficiency drawbacks in schizophrenic patients (Jonsdottir et al., 2021). Similarly, autism shares some of the genetic basis of intelligence, which can explain the relation of the autistic phenotype with the improvement in cognitive performance and convergent thinking, which are essential to creativity (Crespi, 2016). In addition, there may be some relationship between autism, creativity, and artisticality (Kellman, 1998; Spikins et al., 2018).

### Mechanisms associated with personality

The connection between creativity and personality occurs on many levels. For example, creativity, intelligence, openness to experience, and extraversion share a genetic basis (Kandler et al., 2016), which may be a consequence of assortative mating (Conroy-Beam et al., 2019). This genetic basis is involved in dopaminergic systems (Gocłowska et al., 2019). Such systems are activated during process that constitute creative thinking, such as divergent thinking, exploratory activities, and the search for novelty (Vartanian et al., 2018; Gocłowska et al., 2019), commonly associated with individuals high in extroversion and openness (Feist, 2019).

In relation to sex differences, studies have shown that women are more open to experiences than men, whereas others found no gender distinction (Weisberg et al., 2011; Natividade and Hutz, 2015; Schmitt et al., 2017). Differences may be found in the openness to experience subfactors, with women more open than men and men more intellectual than women (Weisberg et al., 2011). Regarding extroversion, women, on average, are more extroverted than men (Weisberg et al., 2011; Natividade and Hutz, 2015; Schmitt et al., 2017).

### Psychological mechanisms: Capacity, perception, and motivation

Creative behaviors may result from psychological structures evolved to solve adaptive problems. Such psychological structures consist of capacities, perceptions, and motivations shaped to generate adaptive outputs (Lewis et al., 2017). This framework has recently been applied to “artisticality” (Varella et al., 2011, 2017; Luoto, 2019a; Varella, 2021) and “musicality” (Bispham, 2009). Here, we propose a similar psychological framework for creativity. That is, creative behaviors of any kind (everyday, academic, performative, scientific, and artistic; Kapoor et al., 2021) will result from evolved psychological structures (creative capacity, aesthetic sense, and motivation) capable of generating cultural creative outputs.

Psychological adaptations are related to intrinsic motivation to perform certain activities. In fact, children and adults from different cultures exercise their creative capacity in games and other activities in search of fun, pleasure, and affective social interactions (Boyd, 2010; Moraes et al., 2022). This creative play may or may not involve aesthetic sensibility (e.g., drawing and painting; Myszkowski et al., 2014, 2018). Further, this exercise of creative and aesthetic capacities is so spontaneous that it does not even need artistic training (Frois and Eysenck, 1995; Boyd, 2010).

In other words, there is motivation early on in development to put creative and aesthetic capabilities into action. Motivation can be defined as organizing and coordinating aspects of behaviors that arise from a wide variety of internal, environmental, and social sources and is manifested at many levels of behavioral and neural organization (Shizgal, 2001). There is intrinsic and extrinsic motivation. Intrinsic motivation is the will or impulse to engage and sustain engagement in a given activity; extrinsic motivation involves engaging in an activity to have rewards external to the activity itself (Ryan and Deci, 2017). People often engage in creative activities because they are intrinsically motivated by the activity, as in the case of involvement with music (Bispham, 2009) and art (Varella, 2021). It is not by chance that individuals who choose art-related courses are more motivated by intrinsic reasons (e.g., using their talents) than extrinsic ones (e.g., parental and media influence, earnings, status; Varella, 2021).

Furthermore, intrinsic motivation did not vary significantly between arts careers (e.g., music, dance, theater, visual arts, and literary studies), suggesting a specific motivational system for general artistic abilities that underlies the expression of all artistic modalities (Varella, 2021). Offering rewards (extrinsic motivation) for creative performance can even decrease the quality of products generated by children (Amabile and Gitomer, 1984) and possibly in other animals, as anecdotally observed in a chimpanzee (Morris, 1962). Motivation in this area appears very early in development (Bispham, 2009; Varella, 2021). Children already draw, and babies are entertained by the mother’s vocalizations and movements, which may indicate the perception of rhythm, fundamental for appreciation and aesthetic productions in music and dance (Trevarthen, 1999; Bispham, 2009).

## Ontogenesis

Some people are more creative than others, which is spontaneously evidenced since childhood (Feist, 2004). As children age, more cognitive maturity ensues, and it may be accompanied by a greater investment of creativity in specific domains, such as visual art, music, literature, or science (Barbot and Tinio, 2015). If creativity has evolved by sexual selection, it is expected that its increase follows changes beginning at puberty, when there is a boost in the release of androgens (Miller, 2000; Miller, 2001). However, that boost is not linear. During puberty, there is a drop in gray matter and the number of dopamine receptors; this likely explains the decrease in the cognitive aspect of creativity and the increase of the aspects related to personality, such as openness, thrill-seeking and novelty (Barbot and Tinio, 2015).

The relationship between androgens and creative expression could indicate the existence of sexual dimorphism in creativity, but, considering physical and psychological characteristics in general, humans have milder sexual dimorphism than other species (Janicke and Fromonteil, 2021). More specifically, humans tend to be more monomorphic because both sexes are heavily invested in offspring and, therefore, more selective in choosing mates (Stewart-Williams and Thomas, 2013). That likely led both females and males to evolve and develop fitness indicators for mate attraction (Stewart-Williams and Thomas, 2013). That would explain the similarity in creativity between men and women and the greater variability in different creative domains in both sexes (details in Table 1; Varella et al., 2017; Nakano et al., 2021).

Evidence on the variability of creativity in childhood is mixed (Lau and Cheung, 2015; He, 2018). There is evidence of greater male variability in adulthood (He and Wong, 2011; He, 2018), which has been replicated in several countries in African (Karwowski et al., 2016a) and European (Karwowski et al., 2016b) continents, as well as the United States (Taylor and Barbot, 2021), with a few exceptions (He et al., 2013; Ju et al., 2015; Lau and Cheung, 2015). For instance, a study conducted in Poland reported greater male variability in all ages in the performance at a task that involved completing a drawing as creatively as possible (Karwowski et al., 2016b). In a second study, various domains of creativity were examined in a sample of people of diverse ages and schooling. There were no sex differences in the means in performance or potential, but there was sex differences on specific domains of creativity (e.g., Karwowski et al., 2016b), as well as a greater male variability (e.g., Karwowski et al., 2016b).

However, the variability in creativity in each sex may also depend on the creative domain evaluated. He (2018) measured divergent thinking and creative problem-solving in Hong Kong university students. Greater male variability was found in divergent thinking tasks involving images but not verbal tasks (He, 2018). See Table 1.

Average creativity between men and women is highly variable among studies, even among creative domains (Abraham, 2016). In childhood, girls perform better than boys (Cheung and Lau, 2010; Hemdan and Kazem, 2019). In adults, most studies show female superiority (45.2%), while others find no sex differences (31.5%; Nakano et al., 2021). In a study on 3–7-year-olds, girls were better represented at the top of creativity distribution, while in the age range of 19–23-year-olds, the boys had the best representation at the top (He et al., 2015). In a longitudinal study that lasted four years in Hong Kong, boys and girls from 8 to 11 years old displayed improvement in creativity, with female superiority (He, 2018), but boys’ creativity increased from age 15 and surpassed that of girls at 16 (He, 2018).

The disparity between men and women may also be perceived in personality traits associated with creativity. For example, teenage girls are more extroverted and open to experiences (personality traits associated with creativity) than boys of the same age, which corroborates the evidence of higher creativity in girls in this age range (De Bolle et al., 2015). These disparities in personality may stem from male and female timing in sexual maturation. Girls enter puberty earlier, which seems to explain their improvement in socialization and cognitive performance ahead of boys (De Bolle et al., 2015).

## Phylogenesis

If creativity is a result of evolution, it is to be expected that there would be similar characteristics in other species (e.g., Cauchard et al., 2013). Likewise, if this process is partly due to sexual selection, it makes sense to assume that creativity plays a role in the reproduction of humans and other species.

### Creativity and innovation in other species

Species that develop in hostile and unstable environments tend to have an intense social life, large brains, extended youth, and an ability to learn; that is to say, these species are marked by higher phenotypic plasticity, which carries advantages in solving adaptive and new problems (Lefebvre, 2013). This is the case with some primates, including *Homo sapiens*, and birds, like crows (Lefebvre et al., 2004; Sanz et al., 2009; Lefebvre, 2013; Bandini and Harrison, 2020). For instance, crows choose hook-shaped twigs, ideal for “fishing” food from hard-to-reach places (Taylor, 2014). Chimpanzees fashion sprigs to feed on termites from inside trunks (Bandini and Harrison, 2020). An anecdotal sample showed that a male chimpanzee used plant leaves and branches to emit specific sounds that caught females’ attention (Bandini and Harrison, 2020).

Animals more inclined to be innovative tend to be neophilic, i.e., they run more risks and seek novelty and sensations (Kaufman et al., 2011). Orangutans illustrate the case of a neophobic primate known for innovating less in natural environments; on the other hand, chimpanzees are more neophilic (van Schaik et al., 2016; Bandini and Harrison, 2020). Just like humans, these plastic behaviors are associated with dopamine in various brain systems (Kaufman et al., 2011).

There has been shown sex differences in innovation. Creativity can be used by the less dominant sex as an alternative sexual and foraging strategy, as it happens among chimpanzees, where females are the most habitual tool users (Reader and Laland, 2001). However, Lefebvre (2013) states that males innovate more than females not only in primates, but also in birds and in ancestral humans. Using tools to enable more accessible food gathering brings evident benefits for survival and less obvious ones for reproduction. Males with privileged access to food gain advantages in the social hierarchy, ensuring access to allies and females (Reader and Laland, 2001).

### Ornamental creativity: The case of bowerbirds

Some displays of creativity are more ornamental or aesthetic than pragmatic, impacting mating (Miller, 2001). The bowerbird is a classic example of an ornamental manifestation of cognition in non-human animals. The male satin bowerbird increases its reproductive success by decorating the bower with blue objects (Borgia, 1986). To do this, males need an aesthetic sense to decorate the bower, just as females need an aesthetic sense to evaluate the best-decorated bower. Depending on the definition of art and aesthetics used, the bowers produced and appreciated by bowerbirds can be considered non-human examples of art (Endler, 2012). This aesthetic sense needs a certain cognitive complexity typically present in altricial species (Boogert et al., 2011).

In fact, females in several non-human animal species seem to prefer males who tend to exhibit better cognitive performance. For instance, males that build the fanciest bowers have greater reproductive success, and these males are better at problem-solving (e.g., getting food from a box; Keagy et al., 2009). Also, female budgerigars remain closer to males that manifest more complex problem-solving (Chen et al., 2019). The same behavior is observed in eastern meadow voles (*Microtus pennsylvanicus*) and North Island robins (*Petroica longipes*; Spritzer et al., 2005; Shaw et al., 2019). These behaviors suggest that males who exhibit greater cognitive complexity tend to attract females. However, research is unclear as to whether exhibiting cognitive behaviors attracts females or whether some other trait, indirectly correlated with cognition, generates that attraction.

### Ornamental creativity in *Homo sapiens*

As an altricial species (or a secondary altricial species, see Portmann, 1969), humans also have a large brain and prolonged development that favor cognitive flexibility. The genus *Homo* seems to have been expressing itself creatively for thousands of years. *Homo erectus* already used pigments 800,000 years ago likely for decorative purposes in Southern Africa; 450,000 years ago, they already scratched arbitrary lines on mollusk shells, just as chimpanzees seem to do; 40,000 years ago, *Homo sapiens* made cave paintings and decorated tools from the American continent to Asia (Høgh-Olesen, 2018).

The advent of decorated tools suggests that technical and ornamental displays of creativity have co-evolved. Tools probably started to be produced to solve practical problems, but ornamenting these tools could have social functions (e.g., identification in the group hierarchy), which characterize weapons and uniforms in more recent human history (Menninghaus, 2019). However, these creative and artistic manifestations may be collective activities of the whole group, not restricted to specialized individuals, i.e., professional artists (e.g., Mackie, 2015; Rabazo-Rodríguez et al., 2017).

A specific (and controversial) hypothesis about Paleolithic tools’ pragmatic and ornamental function is that of the sexy handaxe (Kohn and Mithen, 1999). According to this hypothesis, handaxes would have the same ornamental function as the bowerbird’s decorated bower (Kohn and Mithen, 1999). An effective handaxe just for hunting would not need to be overly symmetrical, big, or heavy, making them difficult to use as a weapon (Menninghaus, 2019). Thus, such artifacts could also have social functions, such as signaling identity and potential as a reproductive partner, which could indicate the level of status, dominance, and/or aesthetic skills. It is also possible that the symmetry of artifacts takes advantage of sensory system biases that make specific patterns more attractive than others (such as more symmetrical faces; Gangestad and Thornhill, 2003). In the aggregate, such findings as reviewed above suggest that displaying aesthetic and ornamental capabilities linked to creativity is not restricted to the present but also to the evolutionary past.

## Function

The available evidence indicates that creative manifestations—mainly ornamental ones, but also pragmatic ones—may play a unique role in attractiveness. But why? In the following sections, we will examine evidence related to various theories (see also Davis and Arnocky, 2022).

### Good genes and mental fitness

According to the good genes model, traits selected by sexual selection confer indirect advantages to the offspring, such as genetic quality (see Davis and Arnocky, 2022). “Providing” good genes is essential in species with low male parental investment (Trivers, 1972; Zahavi, 1975). For example, peacocks display their striking plumage, and bowerbirds display their decorated bowers that serve as honest signals of genetic and/or phenotypic quality (Zahavi, 1975; Borgia, 1986). The mental fitness hypothesis uses the same logic applied to the mind. According to that hypothesis, higher-than-average creativity and intelligence would lead to improved reproductive success by indirectly indicating genetic quality (Miller and Todd, 1998; Miller, 2000, 2001; Karamihalev, 2013). Genetic quality means fewer harmful mutations (Klasios, 2013). These mutations can disrupt the organism’s development, including the brain (Klasios, 2013). Therefore, displaying creative products ultimately indicates that the producer is healthy and has an efficient brain (Miller and Todd, 1998; Klasios, 2013).

#### Genetic quality of creative individuals

If the mental fitness hypothesis is correct, there will be associations between creativity (and related cognitive variables) and indicators of genetic quality, and these indicators may be associated with health (Zahavi, 1975; Miller, 2000). One of the cognitive performance indicators most associated with health is intelligence (possibly the cognitive part of creativity). Higher intelligence is related to longevity and a lower risk of suffering from certain diseases, such as hypertension, heart problems, and Alzheimer’s disease (Arden et al., 2009a, 2015; Deary et al., 2019). That can be explained by the fact that intelligent people have healthier behaviors and would be subject to less risk (Gale et al., 2010).

Biological factors can also explain the relationship between intelligence and health. Deleterious mutations cause instabilities in the organism’s development, impairing brain development and cognitive performance (Gajos and Beaver, 2017). Paternal age is a known indicator of detrimental mutations (Gajos and Beaver, 2017). Children of older fathers are more at risk of having autism, attention-deficit/hyperactivity disorder, bipolar disorder, schizophrenia, and depression (D’Onofrio et al., 2014; Gajos and Beaver, 2017; Woodley of Menie and Kanazawa, 2017). More specifically, older fathers tend to have a little less intelligent children (even controlling for other variables), with a reduction of.84 to 1.23 points in *g* factor for each additional decade in paternal age since conception (Arslan et al., 2014; D’Onofrio et al., 2014; Woodley of Menie, 2015; Gajos and Beaver, 2017).

It is expected that characteristics affected by instabilities in development are interrelated. For instance, developmental disturbances lead to higher bilateral asymmetry in the body; the higher the asymmetry, the lower the IQ (Banks et al., 2010). Genetic mutations that hinder normal development also impact the quality of sperm (Jeffery et al., 2016). One study found a positive correlation between sperm quality and intelligence (Arden et al., 2009b); however, another, more recent one, did not (DeLecce et al., 2020).

Geary (2019) suggested that detrimental mutations that affect cognitive performance involve small inefficiencies in cellular processes. More precisely, genetic problems would affect the workings of cellular organelles, such as mitochondria, in charge of cell energy production and operation of the immune system and the brain; difficulties in cellular energy production would affect neurons, which in turn would impact brain functioning (Geary, 2019). Up to now, this notion seems largely speculative, though promising (see Savi et al., 2020; Ujma and Kovacs, 2020). However, other studies provide little supporting evidence for this hypothesis, while some studies have even reported contradictory evidence. For example, Mosing et al. (2015) showed significant correlations between musical aptitude and genetic quality measures only in females, when one would expect to find this result also in men.

Health and cognitive performance may also be associated in other species. For instance, better cognitive performance in bees often indicates an absence of parasites (Gegear et al., 2006). That happens because fighting infectious agents is as costly as investing in complex cognition; thus, there is a trade-off between immune function and cognitive performance (Boogert et al., 2011). Thus, being healthy, and having good learning and problem-solving skills simultaneously, can reflect high levels of genetic quality, or low parasite load. That is compatible with evidence showing that males who are better at problem-solving and learning and have better inhibitory ability have healthier offspring and are, on average, preferred in mating contexts (Spencer et al., 2005; Minter et al., 2017).

Though cognitive qualities imply health, these qualities may be valuable in the mating market for providing more advantages in obtaining resources that may later increase reproductive success (Stephen and Luoto, 2021). Cauchard et al. (2017) suggest this by showing that males of a bird species with better cognitive performance care more about their offspring. However, this hypothesis does not explain why females of promiscuous species (i.e., in which the females do not need the male’s resources) also mate with males having good cognitive qualities (Borgia, 1986; Keagy et al., 2011, 2012).

#### Variability and sexual dimorphism

Fitness indicators vary more than traits evolved for other functions not related to fitness. This variation is due to the number of genes associated with these traits (pleiotropy), the susceptibility to mutations that affect development, and the sexual selection pressure that selects the trait according to the “more is better” logic (Miller, 2000). The heritability of attributes, manifestations, and achievements in a creative domain in men and women suggests that creativity can be a fitness indicator (details in Table 1).

If creativity is a mental adaptation evolved by sexual selection to indicate fitness potential, then it is expected to show high variability in the population due to the large number of genes involved in the expression of this adaptation (pleiotropy; Miller, 2000). Creativity shows high heritability and high variability in men and women, depending on the creative domain considered (see Miller, 2013; Varella et al., 2017; Table 1).

Yet what role does creativity have in attractiveness? Are there sex differences in the role of creativity in attractiveness? Men seem more interested than women in creative activities, and more engaged than women in creative behaviors in the past, according to a scale used to measure interest in creative activities and creative behaviors (Beaussart et al., 2012). Men are also more creative with unexpected flirting behaviors (White et al., 2018). A recent meta-analysis showed a male advantage in creative performance that the authors attributed to cultural factors (Hora et al., 2021). However, previous reviews found no sex/gender differences in creative ability or creative achievement in general but in specific domains of creativity (Baer and Kaufman, 2008).

Why are men more engaged and prominent in art than women, despite their similar creative performance? Men and women can use their creative potential in different ways. For instance, in comedy women use their creativity more in assessing humor, while men use theirs more in producing humor (Greengross et al., 2020). Similarly, one study mentioned the presence of more women (69%) than men in the front rows of music concerts (Sluming and Manning, 2000).

Sociocultural factors can also play a role in these sex differences. Indeed, men are better represented in artistic fields, but it is also a fact that for most of history, women have had fewer chances and fewer incentives to engage and achieve prominence in art (Varella et al., 2017; Rosenthal and Ryan, 2022). A meta-analysis of cross-cultural studies shows that women are “more artistic,” which suggests that sex differences in engagement in these fields may vary culturally (Ellis et al., 2008). Varella et al. (2010) found that women actually appreciate more than men an unknown instrumental piece of music, that women report to appreciate more music in general than men, and that women also report singing more than men. Another study showed a greater number of women in samples of gifted students in art-related courses (Holahan et al., 1995). Given the divergent findings, further studies are needed to verify whether sex differences concerning artistic manifestations exist and whether are more explainable by psychological or cultural factors (Hora et al., 2021).

#### The influence of creativity on attractiveness

Anecdotal evidence suggests that creativity is attractive. Creative geniuses in art and science (e.g., Lord Byron, Albert Einstein, Pablo Picasso, Van Gogh, and Charles Chaplin) are known for having had many casual sex partners, marriages, and children (Karamihalev, 2013). We have already shown evidence that creativity is universally attractive, but in this section, we will discuss specific evidence in more detail.

It is difficult to say whether this is a causal relationship or merely an association, but studies have shown that, for example, men with a larger artistic output (e.g., poets and painters) have a larger number of sexual partners (Clegg et al., 2011; Beaussart et al., 2012; Lange and Euler, 2014). Mosing et al. (2015) showed that boys had higher music achievement than girls. Furthermore, there was a negative association between sociosexuality, music aptitude and achievement in both sexes (Mosing et al., 2015). The authors indicated that these results are in accordance with the mutual mate choice model, in which both sexes utilize music to attract partners in a long-term reproductive strategy. But other studies show the opposite in terms of reproductive strategy. For instance, female and male poets and painters had more sexual partners (i.e., short-term reproductive strategy) than controls from other non-artistic professions (Nettle and Clegg, 2006). After reading vignettes describing a man with different levels of creativity and resources, women consider creative men with fewer resources as more attractive than less creative men with abundant resources; an limitation of this study was the low number of participants (41 women; Haselton and Miller, 2006). Male faces become more attractive if presented alongside creative text or music (Marin et al., 2017; Watkins, 2017; Marin and Rathgeber, 2022). The attributed attractiveness is even greater when men produce more complex (compared with less complex) musical patterns (e.g., Charlton, 2014; see Bongard et al., 2019). Furthermore, men, more than women, prefer songs with more complex, technical musical patterns and those less present in popular styles of music (Colley, 2008; Ord, 2020). The male preference for complex and technical music may be explained as a consequence of the evolved aesthetic propensities to impress women (which contradict the mutual mate choice model). An alternative explanation is that observed male preference is a byproduct with no signaling component *per se*. Artists and other creative people tend to be more open to experiences than other professionals, and more open people are likely to be more erotically inclined and less sexually restricted (Natividade and Hutz, 2016).

But the artistic propensities of both sexes can be used to attract mates (intersexual selection) and to compete for mates (intrasexual competition; Varella et al., 2022). For example, women propensities to visual and circus arts were related to intersexual selection, while literary and musical arts were related to both elevated inter-and intrasexual selection (Varella et al., 2022). In men, circus arts were related intersexual selection and visual arts with intrasexual competition (Varella et al., 2022).

Everyday displays of creativity also seem to impact attractiveness. For instance, women are attracted by men who can employ metaphors, make them laugh at jokes, and have significant verbal prowess (Lange et al., 2014; Gao et al., 2017; Greengross et al., 2020). Summing up, ornamental signs of creativity seem to be mainly linked to male attractiveness, though some suggest they also increase the attractiveness of creative women (Kaufman et al., 2016). However, some studies contradict the association between creativity, openness, and unrestricted sexuality. For instance, though better musical performance increases attractiveness (Madison et al., 2018), individuals with a more prominent musical output take longer to have their first intercourse, and women have fewer sexual partners as their musical output grows (Mosing et al., 2015).

#### Creativity, attractiveness, and reproductive strategy

In general, costly signaling develops in promiscuous species, in which the only expected male investment is genetic quality. Humans are diversified in their reproductive strategies and may enter short-term relationships in some situations, a kind of partnership akin to the promiscuity of other species (Gangestad and Simpson, 2000; Schmitt, 2005; Buss and Schmitt, 2019). Hence, if creativity signals good genes, it is expected that creative and original people are more attractive for casual relationships, as it seems to occur among bowerbirds (Borgia, 1986; Miller, 2001; Keagy et al., 2009). Findings regarding a preference for creative partners for short-term relationships are inconclusive at best. Some studies propose that women prefer creative men for long-term partnerships (Madison et al., 2018), for short-term ones (Haselton and Miller, 2006; Charlton, 2014; Mosing et al., 2015), or both (Griskevicius et al., 2006; Prokosch et al., 2009).

For instance, Madison et al. (2018) and Charlton (2014) have shown that presenting men with music increased their attractiveness for short-term relationships. Prokosch et al. (2009) recorded men as they performed four activities demanding verbal intelligence and creativity and then showed the videos for women to evaluate them. Intelligence predicted male attractiveness for long-term relationships, while creativity predicted attractiveness for short-and long-term relationships (Prokosch et al., 2009). Similarly, in two studies, male artists had a greater interest in long-term relationships, but one of the studies showed an association between being more successful in the career and the larger quantity of children, a sign of a larger reproductive effort characteristic of short-term relationships (Clegg et al., 2011; Mosing et al., 2015). The attractiveness of creativity in short-and long-term relationships may contradict the relationship between creativity and good genes; but it may also indicate that, precisely because it signals good genes, creativity becomes attractive in long-term relationships, in which people are more demanding (for other evolutionary theories, see Dissanayake, 2008; Luoto, 2019a; Mehr et al., 2021; Savage et al., 2021).

#### Fertile window

If creativity is a kind of costly signaling, creative men will be more desirable to women in the fertile window of the menstrual cycle. The higher chance of getting pregnant at this time would heighten sexual appeal (Stern et al., 2021) and the preference for men with a better genetic constitution (see Thomas et al., 2021). Some evidence confirms that women in their fertile window would rather have casual relationships with creative men (e.g., Charlton, 2014; Marin et al., 2017). Haselton and Miller (2006) showed that women in the fertile window preferred short-term relationships with creative men (regardless of the men’s amount of resources). Furthermore, women were more creative during their fertile phase (Galasinska and Szymkow, 2021; Galasinska and Szymkow, 2022).

#### Lack of reproductive success in psychopathological scenarios

Some psychopathologies may be dysfunctional expressions of ornamental creativity as if the fitness indicator had failed or overshot its optimum. Indeed, people with schizotypal traits attract partners using metaphors and verbal proficiency (Lange et al., 2016; Gao et al., 2017). On the other hand, people with schizophrenia have cognitive and linguistic problems that make it difficult to express such “verbal ornaments” and, therefore, also impair their attractiveness (Shaner et al., 2004). Schizophrenic symptoms peak at puberty, when levels of circulating androgens increase, influencing secondary sexual traits. The increase in symptom severity is related to dopaminergic antagonists, which in humans and other animals are associated with sexuality and reproduction (Shaner et al., 2004; Del Giudice et al., 2010). In addition to being associated with creativity, schizotypal and autistic traits would theoretically be linked to short-and long-term reproductive strategies, respectively (Del Giudice et al., 2010). That may explain the relationship between creativity, personality, and sexual selection. Individuals with an autistic phenotype invest more in offspring and favor long-term relationships (Del Giudice et al., 2014; Ponzi et al., 2016). Autistic creativity is mainly characterized by convergent thinking, exhibiting greater pragmatic creativity. According to Del Giudice et al. (2010), the prevalence of autism is currently linked to the selection of genes that are associated with greater systematization, greater attention to detail, and the ability to innovate in technical fields, which may have become more helpful from the Holocene, a period characterized by agriculture (Harpending and Cochran, 2002). However, attention to detail is also crucial in archeological cave paintings (Kellman, 1998; Spikins et al., 2018). These psychological skills would be worth greater prestige and access to resources in these cultural contexts, also leading to a higher number of mating opportunities for individuals with these skills, particularly men (Henrich and Gil-White, 2001; Del Giudice et al., 2010). That would suggest that the reproductive benefit of this kind of creativity lies in facilitating the acquisition of resources, not necessarily in signaling good genes.

On the other hand, schizotypal individuals may have occupied the role of shamans in traditional societies (Dein and Littlewood, 2011). That would explain ancestral artistic displays as part of religious rituals rather than pure art, the latter being more common in contemporary art (Høgh-Olesen, 2018). Theoretically, artistic skills and a more original personality would improve access to short-term relationships, which is difficult to infer from ancestral societies, yet studying a hunter-gatherer population, Smith et al. (2017) found that the value of good storytellers is reflected in the fact that they also have increased reproductive success and receive more resources than less-skilled storytellers. It is also conceivable that this ornamental creativity associated with schizotypy does not guarantee many privileges in these societies. A study of the Meru, a semi-nomadic tribe in Kenya, showed that more creative people had more resources, although they had fewer children, fewer grandchildren, and fewer wives/husbands (Lebuda et al., 2021). That is the opposite of what is expected from an adaptive perspective, namely that creativity leads to reproductive success. One possible explanation is that the attraction to creative partners is recent in human history, in societies that value disruption, innovation, and rapid change. Traditional societies value stability, traditions, and rules. Or, yet, creativity was adaptive from a sexual selection point of view in our environment of evolutionary adaptedness (EEA), but is not adaptive anymore because societies and environments (including the Meru) have changed so drastically.

#### Context

Socioecological stimuli and contexts exert an important influence on creativity. The stimuli and contexts that inspire men and women to perform more creatively may reveal the influence of sexual selection. For instance, men behave more creatively (produce better creative descriptions of abstract paintings) after seeing photos of attractive women. That suggests that a romantic stimulus activates cognitive/neural mechanisms linked to reproduction, leading men to perform better on tasks that might impress women. The increase in male creativity was maintained even when participants performed creative tasks after imagining themselves having a short-term or long-term relationship (Griskevicius et al., 2006). Nevertheless, female creativity increased in the face of greater assurance that the potential partner would invest in a long-term relationship, which may indicate that creativity was selected in the female sex to deal with a reproductive strategy more focused on attracting investment from partners in a committed relationship (Griskevicius et al., 2006).

This study reinforces the idea that creativity evolved by sexual selection in men and women (Baer and Kaufman, 2008; Varella et al., 2011, 2014, 2017; Miller, 2013). The increase in female creativity, given the possibility of short-and long-term relationships, indicates that ornamental creativity may have evolved for signaling both good genes and an ability to acquire resources.

## Resources

It is common for females to select males based on their ability to contribute resources to offspring (Andersson, 1994; Davis and Arnocky, 2022). Parental care is very important to the human species, and resources are one of the forms of parental investment (Andersson, 1994), which is why men with more access to resources are universally considered more attractive, all other things being equal (Buss and Schmitt, 2019; Walter et al., 2020), regardless of the level of gender equality (Zhang et al., 2019). Indeed, intelligence and creativity are critical to activities that implicate access to resources, e.g., academic and professional activities, increasing attractiveness in modern societies (see Lebuda et al., 2021). Intelligence and creativity may not be attractive in themselves but as markers of resource-related potential. Thus, it is possible that intelligence and creativity are not considered attractive in societies (as may be the case with the Meru) where access to resources does not depend on intelligence or creativity.

A possible counter-argument is that even if creativity is attractive because it increases access to resources, this would not necessarily exclude its possible role as an indicator of good genes (Luoto et al., 2019a). After all, individuals able to obtain resources in a given context must be healthy enough to participate in activities that require physical and cognitive effort. As a consequence, it may be that the more attractive women think they are, the more they prefer men who rank high on signs of health and social status, because women’s attractiveness is a fungible currency on the mating market that can be “exchanged” for traits that women seek (Buss and Shackelford, 2008). Creativity can be a reliable sign of both health and status. This would explain why women prefer creative men for short-term and long-term relationships (e.g., Griskevicius et al., 2006; Prokosch et al., 2009).

### Cognition as a weapon in the struggle for status

According to Winegard et al. (2018), cognitive resources increase attractiveness only when they provide culturally valuable assets. More precisely, inventing new technologies or artistic products confer prestige, which can be “exchanged” for resources and mate partners (Henrich and Gil-White, 2001). This model emphasizes intrasexual competition more than intersexual selection (Winegard et al., 2018). Intrasexual and intersexual competition are two main mechanisms of sexual selection (Darwin, 1871; Puts, 2016). In intrasexual competition, individuals (usually males) compete with others of the same sex for access to the opposite sex, which leads to the evolution of ornaments (e.g., plumage, singing, dancing) and/or weapons (e.g., horns, talons, fangs); whereas in intersexual selection, males mostly display their ornaments and weapons directly to the opposite sex (Berglund et al., 1996). In humans, males and females are involved with intersexual selection and intrasexual competition (see, e.g., Stewart-Williams and Thomas, 2013). It is possible that ornamental creativity evolved to be useful in intrasexual competition as a way of impressing other men, in addition to being useful in intersexual selection (Winegard et al., 2018). Accordingly, Winegard et al. (2018) mention historical, philosophical, and literary treatises written when most women were not able to read. The contents of such treatises do not seem to appeal to female interests (as pointed out by feminist authors; e.g., Irigaray, 1985) since they deal with war, politics, military strategy, and metaphysics. Men who were more prolific in these fields garnered more prestige among other men, securing access to valuable social assets, such as resources and protection for themselves and their families (Henrich and Gil-White, 2001; Winegard et al., 2018). Thus, men with superior cognitive abilities will also tend to rise in social status hierarchies.

This model based on competition for status shows advantages over the “cultural courtship” model championed by Miller (2000). Miller (2013) states that men and women have been shaped by evolution to display mental abilities indicative of genetic quality (see also Stewart-Williams and Thomas, 2013). A possible flaw in this hypothesis is the belief that humans have always chosen their partners freely and individually. Actually, for most of human history, and still in most traditional cultures, marriages are arranged, i.e., the bride’s family, usually fathers and brothers-in-law, interfere in the groom’s choice (Apostolou, 2017). In other words, throughout history, mate selection has been about advertising one’s attractiveness to other men.

Many male physical traits seem to bear on dominance, but not necessarily on attractiveness, such as a male face, beard, and muscles (Puts, 2010). Creativity seems to have its use in intrasexual competition, and lyrics about the male world seem to illustrate that (e.g., Black Sabbath, ACDC, and Metallica); but it is also true that some such cultural expressions are directed toward the opposite sex (e.g., ‘N Sync and Back Street Boys; see Winegard et al., 2018). For example, among professional male guitarists, the time spent playing the instrument positively predicted desire for casual sex with women, and the speed in playing positively predicted a desire to impress other men (DeLecce et al., 2022). Varella et al. (2022) showed that men and women use their artistic propensities in intersexual selection (female-biased) and intrasexual competition (male-biased). Creativity and aesthetic sense also probably participated in the female intrasexual competition since body beautification is universally crucial in female attractiveness (Varella et al., 2017).

## Sensory exploration, “sexy son” and “runaway selection”

The theories of costly signaling and mental fitness (Zahavi, 1975; Miller, 2000, 2001) have become very popular in explaining the existence of abnormal phenotypes in many species, including creative manifestations in humans (Luoto et al., 2019a). However, Darwin (1871) believed something else: for him, sexual selection picked flashy traits for arbitrary aesthetic reasons (Prum, 2012; Davis and Arnocky, 2022). There is evidence that Darwin’s insight was correct. In many species, individuals are selected to mate through a process called sensory exploration (Verpooten and Nelissen, 2012). For instance, female guppies tend to copulate with males with more markedly orange spots around their bodies. That is not so because these males are genetically better, but because their orange spots co-opt the female’s sensory system, shaped by natural selection to find food of the same color (see Verpooten and Nelissen, 2012). This sensory co-optation process may lead to a “runaway selection,” in which each new generation of males develops phenotypes ever more extravagant and unrelated to any underlying aptitude (Prum, 2012).

Showy phenotypes may also arise if they make the offspring attractive (this is known as the “sexy son hypothesis”). In this process, the alleles of the most selective females spread and are inherited by their offspring, as daughters become more selective and sons showier (Prokop et al., 2012). Such dynamics feedback on themselves on the grounds of attractiveness advantage (which is, in this case, more arbitrary than in the selection based on “good genes”). Hence, while sexual selection based on the “sexy son” phenomenon promises more reproductively successful offspring for being more attractive according to arbitrary patterns, selection based on “good genes” provides offspring that are successful because of better health (Prokop et al., 2012).

However, to Miller (2001), the process of sensory exploration (and, one assumes, the benefits based on “sexy son”) would not continue to be arbitrary under all possible scenarios. Miller (2000) has suggested that ornaments would become so intricate that they will come to depend on the expression of ever more genes; at this stage in complexity, pleiotropy would grow, as well as the threshold of genetic quality required to go on sporting an ever costlier phenotype. Furthermore, sexual selection based on sensory exploration should be more common in not very social species, where the first step to mating is finding a partner. However, primates are social species; thus, locating a potential partner is no problem. On the contrary, the hard part is selecting the best option (Miller, 2000; Verpooten and Nelissen, 2012). Thus, if Miller (2000) is correct, genetic quality becomes a part of sexual selection at some point.

What are the implications of this plurality of mechanisms of sexual selection for the evolution of creativity? Creativity may have evolved by viability selection and also by sexual selection (Luoto et al., 2019a), but the details of this transition are uncertain. Creativity may have begun to grow initially due to general cognitive growth, which is useful for problem-solving. Its effects would have then started to affect other traits that are more useful in partner selection, such as the ability to get food (which in Neolithic humans may happen as social status increases: Winegard et al., 2018). The pressure on expanding cognitive performance would have enhanced this ability to the point that it could only be sustained by individuals having at least enough genetic quality to afford its energy costs (Miller, 2000). From a certain point on, practical benefits of cognitive ability do not increase together with the growth in cognitive ability, which is when conspicuous (i.e., ornamental, fruitless) forms of cognitive ability and creativity to exhibit fitness may arise.

Ornamental traits may be an exaptation, i.e., pragmatic creative capacities evolved initially by viability selection and were later co-opted by sexual selection as phylogenetic exaptations related to aesthetic and artistic production and appreciation (Varella et al., 2011; Luoto et al., 2019a). For instance, probably the first handaxes built by human ancestors would have been used to assist in taking down prey and cutting up carcasses; however, they would gradually be co-opted for aesthetic uses, acquiring ornaments and a more symmetrical look (Mithen, 2003). As an extension of this point of view, creative individuals can reap advantages through functional and/or ornamental extended phenotypes that they have created or acquired. For instance, clothes, cars, and houses were invented for practical reasons (e.g., protection, locomotion) and later acquired ornamental functions that signal (and extend) personal attributes, such as interest in certain forms of romantic involvement, intelligence, and creativity (Luoto, 2019a).

The role of culture in creativity may go beyond exaptations or extended phenotypes. Recently, many traits have been studied that would result from gene–culture coevolution (Bender, 2019). Briefly, this evolutionary process is characterized by selecting genes based on cultural pressures. The evolution of the ability to write is an enlightening example. Human beings did not evolve to read and write. Writing is a result of learning specific cultural techniques that co-opt brain areas shaped initially to deal with other adaptive issues (Parkinson and Wheatley, 2015); however, as soon as writing spreads and starts generating social benefits, genes associated with a greater ability to learn to read and write were selected (Overmann, 2016). This process in which learned responses acquire a genetic base is called the Baldwin effect, which has been used to explain complex aspects of human cognition (Baldwin, 2018).

That process may have supported the fast growth in complexity in hominine cognitive abilities (see recent proposals of this idea in the context of music: Podlipniak, 2017, 2021; Mehr et al., 2021; Savage et al., 2021). Neuroplasticity may have allowed learning ornamental techniques that initially aimed at reinforcing intergroup cohesion and establishing some form of individual or group identity (Garofoli, 2015). These distinctive ornaments may have begun to bring reproductive advantages for cultural reasons, which is when Baldwin effect’s ability to produce ever more complex ornaments may have been genetically incorporated. The use of the Baldwin Effect here is admittedly speculative. Further studies are needed to explore the relations between culture, plasticity, genetics, and evolution.

## Discussion

Did creativity evolve by sexual selection? This article aimed to answer this question considering a pluralistic Tinbergian perspective (Tinbergen, 1963; Fitch, 2015) and a nomological network of evidence (Schmitt and Pilcher, 2004; Konner, 2021). That is the most up-to-date and comprehensive review to integrate and organize an interdisciplinary body of evidence to answer this question about creativity. In summary, our findings suggest that sexual selection likely influenced the evolution of creativity (Table 1); however, the sub-process behind this influence is unclear. Creativity has multiple adaptive functions regarding sexual selection (for an example on musicology, see Fitch, 2015). More specifically, creativity may have evolved by sexual selection not only because it indicates good genes, potential to acquire resources, dominance, or because it is a useful ability in the production of artifacts (e.g., bowers, paintings, music) that co-opt sensory biases to attract attention. It is probable that creativity is connected to all of these aspects. Evolutionary psychologists must test hypotheses derived from processes of sexual selection other than good genes and costly signaling (Luoto, 2019a; Davis and Arnocky, 2022).

Evidence related to sex differences supports that both sexes have developed fitness indicators (Miller, 2013; see Table 1). Overall, no evidence was found of average differences in creative ability or achievement between adult men and women (Baer and Kaufman, 2008), but sexual dimorphism shows up when specific creative domains are evaluated (Ellis et al., 2008; Varella et al., 2010; Savage et al., 2015; Greengross et al., 2020; Hora et al., 2021; Nakano et al., 2021). Other evidence suggests greater creativity among women (Varella et al., 2017; Nakano et al., 2021). Women showed higher mean and greater variability, compared with men, in creative tasks involving language; men showed higher mean and greater variability in creative tasks involving figures and drawing (He et al., 2015; Lau and Cheung, 2015; Karwowski et al., 2016a,b; Taylor and Barbot, 2021). Such sexual differences are compatible with those found in academic and professional preferences, in which women predominate in areas that involve language and men, in areas that involve spatial reasoning (e.g., Wright et al., 2015).

Furthermore, greater variability appears positively associated with greater phenotypic plasticity, which has been linked to the action of androgenic and dopaminergic systems (Del Giudice et al., 2018; Janicke et al., 2021; see Table 1). Creativity is related with greater phenotypic plasticity (Feist, 2019), dopaminergic system (Reuter et al., 2006; Runco et al., 2011; Mayseless et al., 2013), and testosterone levels in both sexes (Hassler, 1992; Sluming and Manning, 2000; Crocchiola, 2014). However, paradoxically, testosterone promotes left brain lateralization, but creativity is linked to right lateralization (Beking et al., 2018). Future studies should investigate the possible role of androgens in male and female creativity and the mechanisms that promote this association.

Evidence on the relationship between psychopathology and creativity supports the predictions of the mental fitness theory (Miller and Todd, 1998; Miller, 2001). Disorders such as schizophrenia, bipolarity, and autism seem to be dysfunctional manifestations of schizotypal and autistic phenotypes linked to creativity, possibly due to genetic predisposition, developmental instabilities, and evolutionarily novel lifestyle factors such as low-grade systemic inflammation and chronic stress (Rantala et al., 2021, 2022).

Evidence shows that creativity is sexy: it is considered attractive in potential mating partners, at least in WEIRD societies (Buss, 1989; Li et al., 2011; Kaufman et al., 2016; Souza et al., 2016). Future studies should verify the role that creativity plays in attractiveness in traditional societies (e.g., Lebuda et al., 2021). Furthermore, future studies should verify the theoretical framework (e.g., good genes) that explains the attractiveness and evolutionary function of different types of creativity (e.g., aesthetic and pragmatic).

According to the mental fitness theory, ornamental manifestations of creativity would be more attractive in short-term relationships (e.g., Haselton and Miller, 2006; Charlton, 2014; Mosing et al., 2015) because they indicate good genes. But other studies suggest that ornamental creativity may also evolved to retain partners in a long-term relationship (Varella et al., 2017; Winegard et al., 2018). Alternatively, artistic capacities could have evolved because they are able to produce something that the human mind finds beautiful (Darwin, 1871; Prum, 2012).

## Limitations

Despite making an important contribution to the literature on mating preferences by bringing together multiple studies and interpreting how much they support the hypothesis of the evolution of creativity by sexual selection, there are limitations to this review, which future studies may overcome. The first is that, despite the extent of the review performed here, this article is not a systematic review or a meta-analysis.

A second limitation is the focus on mental fitness theory. This focus resulted from our deliberate choice to consider theories and evidence supporting (or not) the evolution of creativity by sexual selection. It was for this reason that we added a discussion about creativity being attractive because it signals good genes or because it almost always (at least in WEIRD societies) equates to resource acquisition. Future evolutionary research on creativity should compare evidence related to sexual selection and other theories, such as exaptations, co-opted by-product and cultural evolution (Luoto, 2019a). This review has gone a long way toward integrating and enhancing our understanding of ornamental creativity as a possible sexual selected psychological trait.

## Author contributions

FN: main argument, writing, and formatation. JN: main argument and revision. All authors contributed to the article and approved the submitted version.

## Conflict of interest

The authors declare that the research was conducted in the absence of any commercial or financial relationships that could be construed as a potential conflict of interest.

## Publisher’s note

All claims expressed in this article are solely those of the authors and do not necessarily represent those of their affiliated organizations, or those of the publisher, the editors and the reviewers. Any product that may be evaluated in this article, or claim that may be made by its manufacturer, is not guaranteed or endorsed by the publisher.
